# Estimation of tissue‐water linear stopping power ratio of the proton beam from proton density‐weighted MRI

**DOI:** 10.1002/mp.70204

**Published:** 2025-12-27

**Authors:** Puspen Chakraborty, Hidetoshi Saitoh, Junichi Hata, Weishan Chang

**Affiliations:** ^1^ Graduate School of Human Health Sciences Tokyo Metropolitan University Arakawa‐ku Tokyo Japan

**Keywords:** D_2_O‐H_2_O phantom, hydrogen (proton) concentration, MRI‐integrated proton therapy, proton density‐weighted MRI, tissue‐water linear stopping power ratio

## Abstract

**Background:**

In MRI‐integrated radiotherapy, image registration between magnetic resonance imaging (MRI) and computed tomography (CT) can introduce systematic errors of up to 5 mm. To avoid such errors in proton therapy, one of the prerequisites is the determination of the tissue‐water linear stopping power ratio $S_{\mathrm{tissue},\;\mathrm{water}}$ from MRI for treatment planning.

**Purpose:**

$S_{\mathrm{tissue},\;\mathrm{water}}$ depends on the elemental composition of the medium. Proton density‐weighted MRI measures the concentration of hydrogen (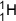), which is independent of the magnetic field strength. This study evaluated the potential of proton density‐weighted MRI to estimate $S_{\mathrm{tissue},\;\mathrm{water}}$ in MRI‐integrated proton therapy.

**Methods:**

Based on ICRU 46, we analyzed and modeled the relationship between tissue‐water hydrogen concentration ratio $H_{tissue,\;\mathrm{water}}$ and $S_{tissue,\;\mathrm{water}}$ at 100 MeV proton energy using linear regression. The model was evaluated for the accuracy of the regression fit and tissue composition variability at proton energies from 70–230 MeV. To assess the accuracy of proton density‐weighted MRI, we developed a deuterium oxide (D_2_O)–water (H_2_O) phantom that replicates the hydrogen concentration range in human tissues. Fast spin‐echo (FSE) and gradient‐echo (GRE)‐based sequences were compared. Positional and voxel‐level signal variability were investigated.

**Results and Discussion:**

For soft and bone tissues, there was a linear correlation (*R* ^2^ = 0.99) between $H_{tissue,\;\mathrm{water}}$ and $S_{tissue,\;\mathrm{water}}$. The inflated lung deviated from this correlation because it includes air volume, which reduces $H_{tissue,\;\mathrm{water}}$ and $S_{tissue,\;\mathrm{water}}$ significantly. By incorporating air and compressed lung from ICRP 110, a linear correlation (*R* ^2^ = 1.00) was found for lung‐related tissues. The D_2_O–H_2_O phantom covered the hydrogen concentration range ($H_{solution,\;\mathrm{water}}$ = 0.30–1.00) relevant to human tissue. A partial molar volume effect in the phantom emphasized the need for mass density measurement. The FSE sequence provided higher image quality and demonstrated a strong linear correlation (*R* ^2 ^= 1.00) between $H_{solution,\;\mathrm{water}}$ and signal‐noise ratio (SNR). By contrast, the GRE showed distortion artifacts. Based on the evaluation criteria, composite uncertainties were 6.89%, 3.00%, and 1.92% for soft, bone, and lung tissues, respectively. Adipose tissue contributed significantly to soft tissue uncertainties.

**Conclusions:**

The relationship between $H_{tissue,\;\mathrm{water}}$ and $S_{tissue,\;\mathrm{water}}$ remained consistent despite variability in tissue composition and treatment energies. The D_2_O–H_2_O phantom, which is simple and reproducible, proved effective for accurately calibrating proton density‐weighted MRI against $H_{tissue,\;\mathrm{water}}$. These findings demonstrate the potential of proton density‐weighted MRI to directly estimate $S_{tissue,\;\mathrm{water}}$. A separate method to identify adipose through lipid concentration measurement may further improve accuracy in soft tissues.

## 1 INTRODUCTION

Proton therapy has the distinct advantage of depositing most of its energy within the target volume using the Bragg peak. To fully utilize this asset, accurate prediction of the proton range is essential.^1^ For treatment planning, the pencil beam algorithm, which requires the tissue‐water linear stopping power ratio $S_{tissue,\;\mathrm{water}}$ to determine the proton range, is typically employed. Conventional methods for deriving $S_{tissue,\;\mathrm{water}}$ rely on computed tomography (CT).^2^ Magnetic resonance imaging (MRI) has recently gained prominence in radiotherapy because of its superior soft‐tissue contrast, radiation‐free imaging, and ability to provide biological information.^3^, ^4^ Image registration between MRI and CT is performed to integrate MRI into the radiotherapy workflow. However, image registration can result in systematic errors of up to 5 mm.^5^, ^6^, ^7^, ^8^, ^9^ To avoid such errors in MRI‐integrated radiotherapy, treatment planning should rely solely on MRI. One of the prerequisites of proton therapy is the determination of $S_{tissue,\;\mathrm{water}}$ using MRI.

Maspero et al.^10^ employed commercially available software MRCAT (Philips Healthcare, Vantaa, Finland)^11^ to generate substitute CT (sCT) for proton therapy. This software utilizes T1‐weighted Dixon MRI,^12^ with a constrained shape model to estimate body contour and segment bone structures. Soft tissues are classified as muscle or fat based on water–fat intensity, while cortical bone and spongiosa are classified according to in‐phase MRI intensities. Predetermined Hounsfield Unit (HU) values are assigned. To improve the voxel‐wise sCT generation method^13^, Guerreiro et al.^14^ applied individual MRI to HU conversion models of the lungs, soft tissues, and bones. As the T2‐weighted MRI used in this study could not capture the lungs and cortical bone, atlas‐based segmentation was performed. Although these studies demonstrated the feasibility of generating sCT, they revealed some limitations: (i) the inability to distinguish individual tissue types, (ii) disregard for intra or inter‐patient HU variations, (iii) requirement for segmentation, and (iv) dependence on the magnetic field strength.^10^, ^13^, ^14^

The estimation of $S_{tissue,\;\mathrm{water}}$ using sCT involves generating sCT and applying a stoichiometric method.^2^ Yang et al.^15^ estimated the uncertainties in $S_{tissue,\;\mathrm{water}}$ using the stoichiometric method. Consequently, incorporating the sCT generation process into the stoichiometric method increases uncertainty. Therefore, a direct MRI to $S_{tissue,\;\mathrm{water}}$ estimation method is favorable. With the same purpose, Gao et al.^16^ employed a deep learning‐based approach that achieved high accuracy for specific tissues; however, it exhibited training data limitations and could not define a wide range of tissue types. In addition, the differences between the phantom‐based training data and actual tissues introduced further uncertainties.

$S_{tissue,\;\mathrm{water}}$ depends on the elemental composition of the medium. Proton density‐weighted MRI measures the concentration of hydrogen (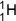) by detecting signals from its nuclei (protons). Note that while the term “proton density (cm^−3^)” is commonly used, “hydrogen (proton) concentration (cm^−3^)” is more precise, as MRI does not provide information about the mass density *ρ* (g cm^−3^). In addition, hydrogen concentration is independent of the magnetic field strength, making it a reliable parameter for different MRI systems. Therefore, this study aims to evaluate the potential of proton density‐weighted MRI for the estimation of $S_{tissue,\;\mathrm{water}}$. To this end, we examined the relationship between the tissue‐water hydrogen concentration ratio $H_{tissue,\;\mathrm{water}}$ and $S_{tissue,\;\mathrm{water}}$ for various human tissues based on the International Commission on Radiation Units and Measurements (ICRU) report 46.^17^ To assess the accuracy of proton density‐weighted MRI in measuring hydrogen concentrations, we developed a deuterium oxide (D_2_O)–water (H_2_O) phantom that replicates the hydrogen range found in the human body.^17^

## 2 MATERIALS AND METHODS

### 2.1 Modeling the relationship between $H_{tissue,\;\mathrm{water}}$ and $S_{tissue,\;\mathrm{water}}$

To calculate $H_{tissue,\;\mathrm{water}}$ and $S_{tissue,\;\mathrm{water}}$ for various human tissues, we utilized the mass density *ρ* (g cm^−3^), weight ratio of hydrogen $w_{H}$ (%), and mass stopping power *S/ρ* (MeV cm^2^ g^−1^) at 100 MeV proton energy from the ICRU 46.^17^ This energy was selected according to Yang et al.^15^ to minimize the range uncertainty caused by treatment planning systems, which assume a constant energy for $S_{tissue,\;\mathrm{water}}$ estimation. To avoid significant age‐related variations in tissue composition, we focused only on adult body tissues. Data on macromolecules (e.g., proteins), MRI‐invisible tissues (e.g., cell nuclei), abnormal tissues or calculi (e.g., lipomas or urinary stones), and unhealthy tissues (e.g., cirrhotic liver) were excluded. Red and yellow marrow compositions were omitted because these tissues cannot be accurately differentiated from the surrounding bone tissues at the clinical image resolution. The reported bone tissue composition is a mixture of cortical tissue and bone marrow.^18^, ^19^, ^20^ Breast tissue composition was described as including only water and lipid. However, the updated International Commission on Radiological Protection (ICRP) publication 110^21^ states that breast tissue comprises adipose and mammary glands that contain proteins. This component is not mentioned in ICRU 46.^17^ Therefore, we excluded the breast tissue from our analysis. Following ICRP 110,^21^ adipose and mammary glands can be used to represent the composition of breast tissue. For adipose and mammary gland tissue compositions, for which a range of samples were available, the mean values were considered. A total of 34 different tissue samples were used in this study. $H_{tissue,\;\mathrm{water}}$ was calculated using the following equation:

(1) $$H_{tissue,\;\mathrm{water}}=\frac{{\left\{\left(w_{H}/100\right)\times \rho \right\}}_{tissue}}{{\left\{\left(w_{H}/100\right)\times \rho \right\}}_{\mathrm{water}}}\;$$

where $w_{H}$ (%) denotes the weight ratio of hydrogen, expressed as a percentage by mass in ICRU 46^17^, and *ρ* denotes the mass density (g cm^−3^). $S_{tissue,\;\mathrm{water}}$ was calculated as follows:

(2) $$S_{tissue,\;\mathrm{water}}=\frac{{\left\{\left(S/\rho \right)\times \rho \right\}}_{tissue}}{{\left\{\left(S/\rho \right)\times \rho \right\}}_{\mathrm{water}}}\;$$

where *S/ρ* denotes the mass stopping power (MeV cm^2^ g^−1^). Table 1 lists the selected tissue samples and the calculated values of $H_{tissue,\;\mathrm{water}}$ and $S_{tissue,\;\mathrm{water}}$. To model the relationship between $H_{tissue,\;\mathrm{water}}$ and $S_{tissue,\;\mathrm{water}}$, linear regression was performed.

**TABLE 1:** Tissue samples and calculated values of $H_{tissue,\;\mathrm{water}}$ and $S_{tissue,\;\mathrm{water}}$ derived from ICRU 46.^17^

#	Tissue samples (Adult)	$H_{\mathrm{tissue},\;\mathrm{water}}$	$S_{\mathrm{tissue},\;\mathrm{water}}$	#	Tissue samples (Adult)	$H_{\mathrm{tissue},\;\mathrm{water}}$	$S_{\mathrm{tissue},\;\mathrm{water}}$
1	Adipose	0.967	0.970	18	Ovary	0.984	1.044
2	Blood (whole)	0.965	1.051	19	Urinary bladder (empty)	0.975	1.034
3	Brain (whole)	0.994	1.037	20	Urinary bladder (filled)	0.993	1.026
4	GI tract	0.975	1.027	21	Ribs (whole)_2^nd,^ 6^th^	0.806	1.315
5	Heart (empty)	0.975	1.044	22	Ribs (whole)_10^th^	0.760	1.397
6	Heart (blood filled)	0.975	1.053	23	Spongiosa	0.896	1.144
7	Kidney	0.966	1.043	24	Cartilage	0.943	1.079
8	Liver	0.965	1.051	25	Cranium (whole)	0.719	1.462
9	Lung (inflated)	0.239	0.258	26	Femur (whole)_30 years	0.831	1.255
10	Lung (congested)	0.975	1.034	27	Humerus (whole)	0.782	1.352
11	Muscle	0.956	1.041	28	Mandible (whole)	0.690	1.514
12	Pancreas	0.984	1.039	29	Sacrum (whole)_Male	0.852	1.226
13	Spleen	0.975	1.053	30	Sacrum (whole)_Female	0.819	1.300
14	Skin	0.973	1.081	31	Vertebra (whole)_C4	0.799	1.322
15	Mammary gland	0.965	1.024	32	Vertebra (whole)_D6, L3	0.831	1.255
16	Thyroid	0.975	1.044	33	Cortical bone	0.583	1.691
17	Testis	0.984	1.036	34	Water	1.000	1.000

### 2.2 Development of a D_2_O‐H_2_O phantom

To clarify the relationship between hydrogen concentration and proton density‐weighted MRI, we developed a D_2_O‐H_2_O phantom containing a hydrogen range identical to that of the human body.^17^, ^22^ D_2_O was selected as a solute because deuterium has a different gyromagnetic ratio (6.54 MHz / T), which makes it invisible in MRI, compared to hydrogen (42.58 MHz / T)^23^. Moreover, D_2_O is nonhazardous and miscible with H_2_O.^24^ Miscibility is essential for creating extremely low hydrogen concentrations that resemble inflated lung tissues. We prepared 10 solutions with varying weight ratios of H_2_O and D_2_O to create a solution‐water hydrogen concentration ratio $H_{solution,\;\mathrm{water}}$, ranging from approximately 0.239 (inflated lung) to 1.00 (water), as shown in Table 1. The weight ratio of H_2_O, $w_{H_{2}O}$ (%), was calculated as follows:

(3) $$w_{H_{2}O}=\frac{m_{H_{2}O}\;}{m_{H_{2}O}+m_{D_{2}O}}\times 100$$

where $m_{H_{2}O}$ and $m_{D_{2}O}$ represent the masses (g) of H_2_O and D_2_O, respectively. For each solution, $m_{H_{2}O}$ and $m_{D_{2}O}$ were accurately measured using a high‐precision Shimadzu analytical balance AUX‐220 (Shimadzu Corporation, Kyoto, Japan). The weight ratio of D_2_O, $w_{D_{2}O}$ (%), was similarly calculated.

To estimate $H_{solution,\;\mathrm{water}}$ using Equation (1), the weight ratio of hydrogen $w_{H}$ (%), was calculated as follows:

(4) $$w_{H}=\frac{w_{H_{2}O}\times N_{\mathrm{mol}}\times M_{H}\;}{\left(w_{H_{2}O}\times M_{H_{2}O}\right)+\left(w_{D_{2}O}\times M_{D_{2}O}\right)}\times 100$$

where $N_{\mathrm{mol}}$ represents the amount of substance of hydrogen in 1 mol of H_2_O; $M_{H}$, $M_{H_{2}O}$, and $M_{D_{2}O}$ represents the molar mass $\left(g\;\mathrm{mo}l^{-1}\right)$ of hydrogen, H_2_O, and D_2_O, respectively. Because D_2_O–H_2_O solutions are mixtures of two different liquids, the mass density $\rho_{solution}$ (g cm^−3^) may not be accurately calculated unless the partial molar volume is known.^25^ Molecular interactions may change the total volume from the sum of the individual volumes, which can change $\rho_{solution}$ and influence $H_{solution,\;\mathrm{water}}$. No previous studies have reported on the partial molar volume effect in D_2_O‐H_2_O solutions. Therefore, to avoid uncertainties, we measured the mass $m_{solution}$ and volume $V_{solution}$ for each solution using the balance mentioned above and a volumetric cylinder, respectively. $m_{solution}$ was divided by the corresponding $V_{solution}$ to estimate $\rho_{solution}$, which was then used to calculate $H_{solution,\;\mathrm{water}}$. To demonstrate whether a partial molar volume effect exists in D_2_O‐H_2_O solutions, the measured $\rho_{solution}$ were compared as a function of $w_{H}$ (%) with calculated $\rho_{solution}$ using the following equation:

(5) $$\rho_{solution}=\frac{w_{H_{2}O}+w_{D_{2}O}}{\left(w_{H_{2}O}/\rho_{H_{2}O}\right)+\left(w_{D_{2}O}/\rho_{D_{2}O}\right)}$$

where $\rho_{H_{2}O}$ and $\rho_{D_{2}O}$ represent the measured mass densities (g cm^−3^) of H_2_O and D_2_O, respectively.

**FIGURE 1:**
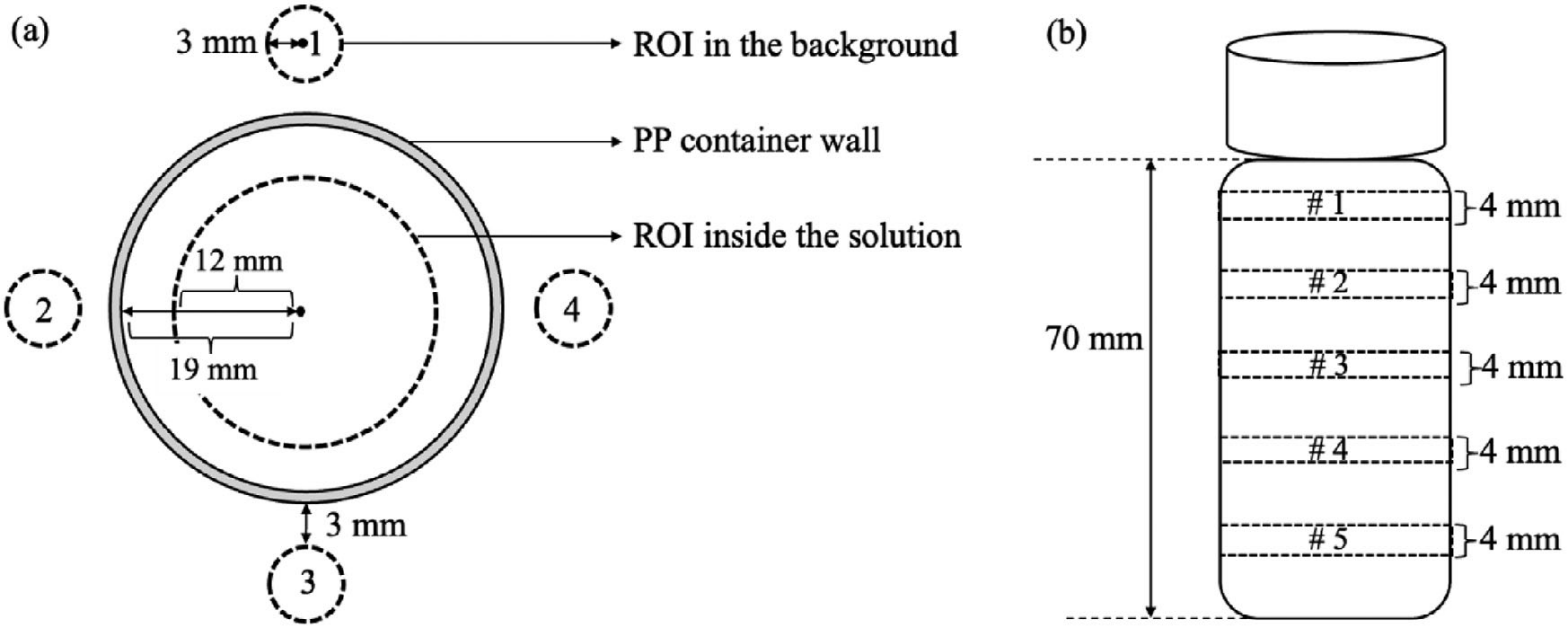
(a) Calculation of signal‐noise ratio (SNR) for the D_2_O‐H_2_O solutions following the National Electrical Manufacturers Association (NEMA) guidelines;^27^ (b) *SNR* estimated at five different depths and averaged to improve accuracy.

The solutions were placed inside polypropylene (PP) containers with rigid body structures and low water vapor transmission rates.^26^ The inner radius of the PP containers was 19 mm, and their height was 77 mm. A hexagonal acrylic frame was used to fix the containers and ensure positional reproducibility. The phantom was scanned using fast spin‐echo (FSE) and gradient‐echo (GRE)‐based proton density‐weighted MRI with a SIGNA Premier 3T scanner (GE Healthcare, U.S.). These image sequences were compared to determine which provided higher accuracy. Details of the imaging parameters are presented in Table 2. Image analysis was performed using Micro‐Dicom software (Version 2023.1.1). A region of interest (ROI) with a 12 mm radius was set to measure the mean voxel value $\bar{I}$ of each solution. This radius covers a substantial area of the solution while minimizing artifacts from the container wall. To analyze the signal intensity relative to the background noise, the signal‐noise ratio (SNR) was calculated using “Method 4” following the National Electrical Manufacturers Association (NEMA) guidelines.^27^ We set four small ROIs, each with a 3 mm radius, on the background 3 mm outside each PP container wall, as shown in Figure 1a. *SNR* was calculated as follows:

(6) $$SNR=\frac{{\bar{I}}_{\mathrm{solution}}}{{\bar{I}}_{\mathrm{background}}}$$

where ${\bar{I}}_{\mathrm{solution}}$ and ${\bar{I}}_{\mathrm{background}}$ denote the mean voxel values of the solution and background, respectively. This process was repeated for five different slices, as shown in Figure 1b, and the results were averaged to improve accuracy.

**TABLE 2:** Parameters used to acquire fast spin‐echo and gradient‐echo‐based proton density‐weighted MRI.

Imaging parameters	Fast spin‐echo	Gradient‐echo
Repetition time (TR)	1800 ms	500 ms
Echo time (TE)	5.87 ms	3.20 ms
Flip angle	111°	30°
Echo train length	8	1
Slice thickness	4 mm	
Field of view	240 mm × 240 mm	
Acquisition matrix	256 × 256	
Voxel size	0.94 mm × 0.94 mm × 4.00 mm	

### 2.3 Uncertainty analysis

#### 2.3.1 Estimation of $S_{tissue,\;\mathrm{water}}$ using the linear regression model relating $H_{tissue,\;\mathrm{water}}$ and $S_{tissue,\;\mathrm{water}}$

The model was evaluated based on three criteria: (1) accuracy of the linear regression fit, (2) variability in tissue composition, and (3) sensitivity to proton energy variation. Uncertainties were derived following the National Institute of Standards and Technology (NIST) Technical Note 1297.^28^

The accuracy of the linear regression fit was assessed by applying the model to estimate $S_{tissue,\;\mathrm{water}}$ for the tissue samples listed in Table 1. The samples were grouped into soft and bone tissues. Uncertainty from the regression fit was categorized as Type A ${\left(u_{A}\right)}_{\mathrm{fit}}$ and calculated as the root mean square (RMS) of the relative differences $\delta_{i}$ between ${\left(S_{tissue,\;\mathrm{water}}\right)}_{\mathrm{est}}$ and ${\left(S_{tissue,\;\mathrm{water}}\right)}_{\mathrm{cal}}$, using the following equation:

(7) $$\begin{array}{cc} {\left(u_{A}\right)}_{\mathrm{fit}} & =\sqrt{\frac{1}{N}\sum_{i=1}^{N}{\delta_{i}}^{2}}\\ & =\sqrt{\frac{1}{N}\sum_{i=1}^{N}{{\left[\frac{{\left(S_{\mathrm{tissue},\;\mathrm{water}}\right)}_{\mathrm{est}}-{\left(S_{\mathrm{tissue},\;\mathrm{water}}\right)}_{\mathrm{cal}}}{{\left(S_{\mathrm{tissue},\;\mathrm{water}}\right)}_{\mathrm{cal}}}\times 100\right]}_{i}}^{2}} \end{array}$$

where *N* is the number of tissue samples i; ${\left(S_{tissue,\;\mathrm{water}}\right)}_{\mathrm{est}}$ and ${\left(S_{tissue,\;\mathrm{water}}\right)}_{\mathrm{cal}}$ represent linear stopping power ratios estimated from the model and calculated as described in Section 2.1, respectively.

To evaluate the model across variability in tissue composition, $S_{tissue,\;\mathrm{water}}$ was estimated for samples obtained from other references.^18^, ^21^, ^29^ Soft tissues of the same type (e.g., adipose) with compositions that commonly vary among individuals, were obtained from Woodard and White.^18^ Other soft tissue compositions that differed from or were not reported in ICRU 46^17^, such as prostate, were sourced from ICRP 110.^21^ For bone tissues, samples were collected from ICRP 110^21^ and Hough et al.,^29^ which provide different compositions for cortical bone and a wide variety of spongiosa compositions. No alternate tissue composition was available for the inflated lung. A total of 105 tissue samples were collected.^18^, ^21^, ^29^ Uncertainty from variability in tissue composition was categorized as Type B ${\left(u_{B}\right)}_{\mathrm{comp}}$ and calculated as follows:

(8) $${\left(u_{B}\right)}_{\mathrm{comp}}=\left(\frac{\delta_{j,\max}-\delta_{j,\min}}{2}\right)/\sqrt{3}$$

where, $\delta_{j,\max}$ and $\delta_{j,\min}$ are the maximum and minimum relative differences, respectively, for each tissue group. Relative differences $\delta_{j}$ between ${\left(S_{tissue,\;\mathrm{water}}\right)}_{\mathrm{est}}$ and ${\left(S_{tissue,\;\mathrm{water}}\right)}_{\mathrm{cal}}$ were calculated using Equation (7), where *N* denotes the number of tissue samples *j*. As the mass stopping power *S/ρ* is not given for these tissue samples, ${\left(S_{tissue,\;\mathrm{water}}\right)}_{\mathrm{cal}}$ were calculated from tissue composition using the Bethe–Bloch equation^2^, ^15^.

To assess the uncertainty from proton energy variation, criteria (1) and (2) were evaluated at 70, 100, 150, and 230 MeV, representing the energy range commonly used in proton therapy.^30^ The tissue compositions used in this study are presented in Supplementary Material 1.

#### 2.3.2 Measurement of *SNR* via phantom repositioning

**FIGURE 2:**
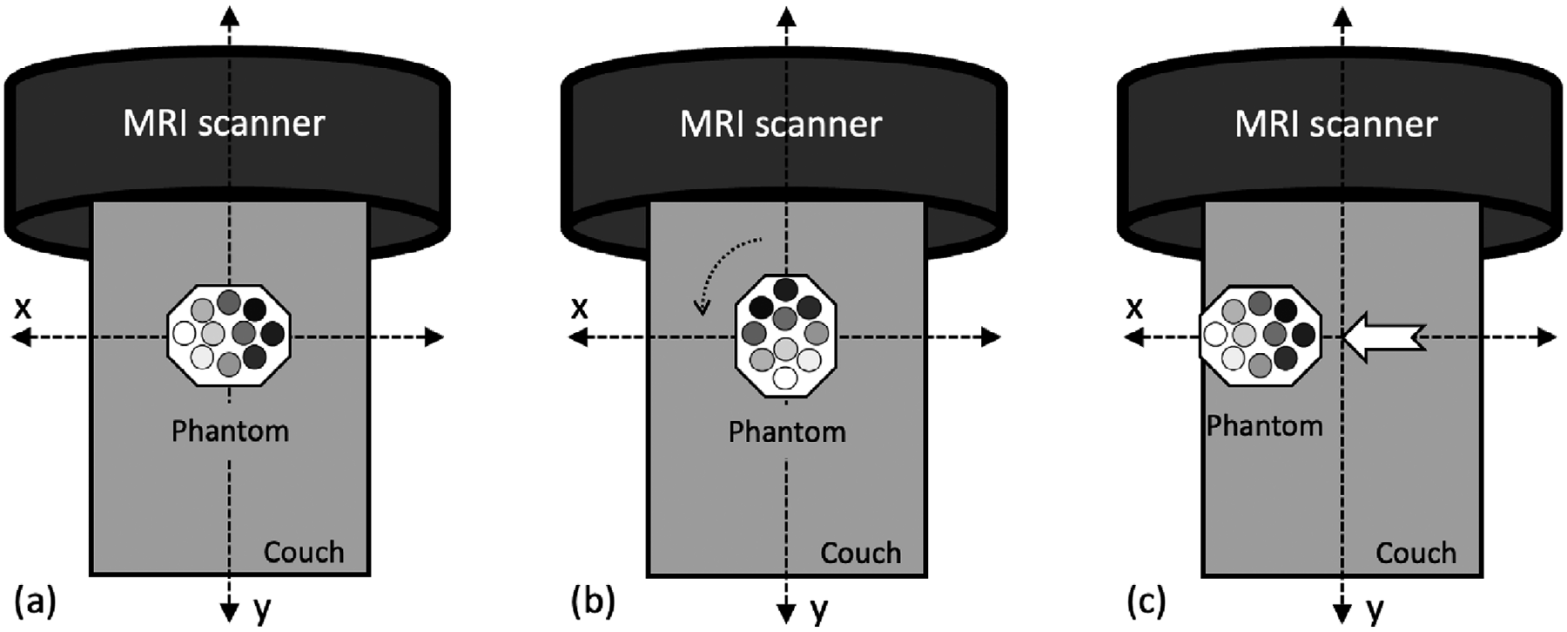
Phantom set up in three different orientations to quantify positional uncertainty in signal‐noise ratio (SNR): (a) primary position, (b) 90° rotation, and (c) edge of the couch position.

To quantify the positional uncertainty in *SNR*, the phantom was rotated by 90° to alter the position of each solution and repositioned at the edge of the couch, as shown in Figure 2. In each orientation, the phantom was scanned using the image sequence determined from the comparison between the FSE and GRE‐based proton density‐weighted MRI. Uncertainties due to positional variation were categorized as Type A ${\left(u_{A}\right)}_{\mathrm{pos}}$ and derived using Equation (7) by calculating the RMS of the relative differences $\delta_{k}$ between $SNR_{\mathrm{arb}}$ at arbitrary positions and $SNR_{\mathrm{pri}}$ at the primary position, where *N* denotes the number of D_2_O‐H_2_O solutions *k*.

## 3 RESULTS

### 3.1 Relationship between $H_{tissue,\;\mathrm{water}}$ and $S_{tissue,\;\mathrm{water}}$

**FIGURE 3:**
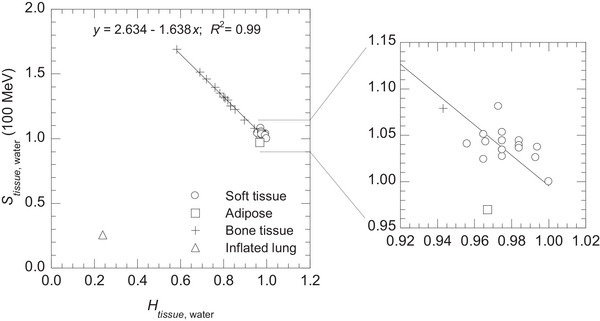
Relationship between tissue‐water hydrogen concentration ratio $H_{tissue,\;\mathrm{water}}$ and linear stopping power ratio $S_{tissue,\;\mathrm{water}}$ at 100 MeV proton energy for 34 tissue samples based on ICRU 46.^17^

Figure 3 shows the relationship between $H_{tissue,\;\mathrm{water}}$ and $S_{tissue,\;\mathrm{water}}$ at 100 MeV proton energy for the tissue samples listed in Table 1. Soft and bone tissues showed a linear correlation with a coefficient of determination (*R* ^2^) of 0.99. In contrast, the inflated lung was allocated to a different area because of its lower $H_{tissue,\;\mathrm{water}}$ and $S_{tissue,\;\mathrm{water}}$, resulting from the air volume within the lung.

Bone tissues were closely aligned with the linear regression line. However, the soft tissues were scattered. Adipose tissue showed the largest difference in $S_{tissue,\;\mathrm{water}}$ from the linear regression line.

### 3.2 Relationship between $H_{solution,\;\mathrm{water}}$ and *SNR*

**FIGURE 4:**
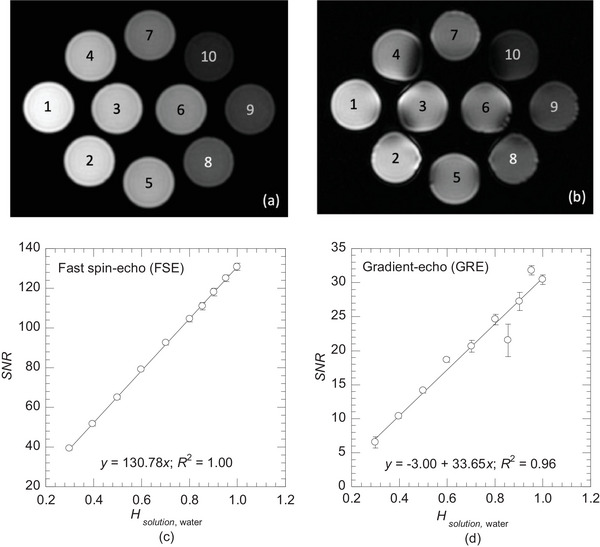
(a) Fast spin‐echo (FSE) and (b) gradient‐echo (GRE)‐based proton density‐weighted MRI of the D_2_O‐H_2_O phantom; The correlation between solution‐water hydrogen concentration ratio $H_{solution,\;\mathrm{water}}$ and signal‐noise ratio (SNR) for (c) FSE and (d) GRE; The error bars represent the standard deviation of the mean voxel values ${\bar{I}}_{\mathrm{solution}}.$

Table 3 presents the composition of the D_2_O‐H_2_O phantom and the *SNR* for the FSE and GRE‐based proton density‐weighted MRI. $H_{solution,\;\mathrm{water}}$ ranges from 0.30 to 1.00, which closely approximates $H_{tissue,\;\mathrm{water}}$ (Table 1); this was achieved by gradually decreasing $w_{H_{2}O}$ (%) and increasing $w_{D_{2}O}$ (%). Figure 4a,b present the FSE and GRE‐based MR images, respectively. The FSE‐based image provides a higher quality, whereas the GRE‐based image shows distortion artifacts. Figure 4c,d illustrate the relationships between $H_{solution,\;\mathrm{water}}$ and *SNR* for these image sequences. A strong linear correlation was observed with *R* ^2^ = 1.00 in the FSE‐based sequence, whereas the *SNR* were scattered in the GRE‐based sequence (*R* ^2^ = 0.96). The error bars represent the standard deviation of ${\bar{I}}_{\mathrm{solution}}$.

**TABLE 3:** Composition of the D_2_O‐H_2_O phantom, including solution‐water hydrogen concentration ratio $H_{solution,\;\mathrm{water}}$; weight ratios of mixture components $w_{H_{2}O}$ (%) and $w_{D_{2}O}$ (%); and signal‐noise ratio (SNR) for the fast spin‐echo (FSE) and gradient‐echo (GRE)‐based proton density‐weighted MRI.

				*SNR*	
#	$H_{solution,\;\mathrm{water}}$	$w_{H_{2}O}$ (%)	$w_{D_{2}O}$ (%)	FSE‐based	GRE‐based
1	1.00	100.00	0.00	130.78	30.43
2	0.95	95.09	4.97	124.98	31.79
3	0.90	89.94	10.06	118.14	27.23
4	0.85	84.98	15.02	110.93	21.53
5	0.80	79.99	20.01	104.49	24.58
6	0.70	70.17	29.83	92.48	20.65
7	0.60	60.00	40.00	79.11	18.65
8	0.50	50.08	49.92	64.95	14.15
9	0.40	39.78	60.22	51.53	10.36
10	0.30	29.95	70.05	39.27	6.53

**FIGURE 5:**
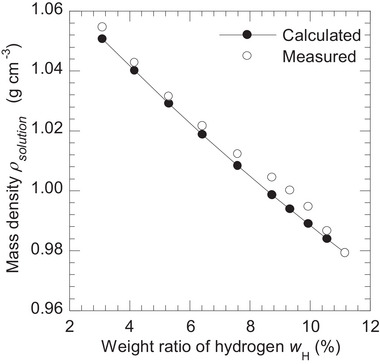
Comparison of the calculated and measured mass density $\rho_{solution}$ (g cm^−3^) as a function of weight ratio of hydrogen $w_{H}$ (%) to represent the partial molar volume effect in D_2_O‐H_2_O solutions.

Figure 5 illustrates the comparison of calculated and measured $\rho_{solution}$ (g cm^−3^) as a function of $w_{H}$ (%). The difference between the calculated and measured $\rho_{solution}$ indicates the presence of partial molar volume effect in D_2_O–H_2_O solutions.

### 3.3 Uncertainty in the estimated $S_{tissue,\;\mathrm{water}}$

**FIGURE 6:**
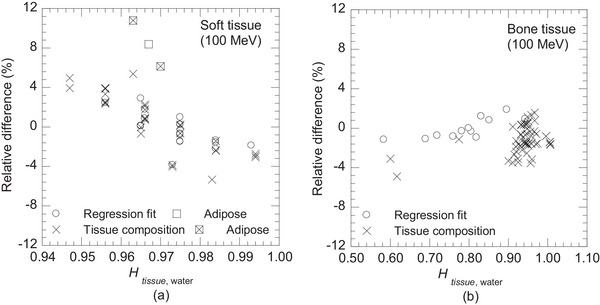
Relative differences (%) between ${\left(S_{tissue,\;\mathrm{water}}\right)}_{\mathrm{est}}$, estimated using the linear regression model, and ${\left(S_{tissue,\;\mathrm{water}}\right)}_{\mathrm{cal}}$, calculated from tissue composition for (a) soft and (b) bone tissues, while analyzing the accuracy of the regression fit and variability in tissue composition.

Table 4 presents uncertainty in the estimated $S_{tissue,\;\mathrm{water}}$ using the linear regression model relating $H_{tissue,\;\mathrm{water}}$ and $S_{tissue,\;\mathrm{water}}$. At 100 MeV proton energy, uncertainty from the regression fit ${\left(u_{A}\right)}_{\mathrm{fit}}$ was 2.51% for soft tissues and 0.94% for bone tissues. Uncertainty due to tissue composition variability ${\left(u_{B}\right)}_{\mathrm{comp}}$ was 4.60% and 1.73% for soft and bone tissues, respectively. The relative differences (%) are illustrated in Figure 6. Across the 70–230 MeV energy range, uncertainty variations remained within 0.14% for all tissues. Because no alternative tissue compositions were available for the inflated lung, this tissue was excluded from the analysis.

**TABLE 4:** Uncertainty in the estimated tissue‐water linear stopping power ratio $S_{tissue,\;\mathrm{water}}$ using the linear regression model, arising from the regression fit ${\left(u_{A}\right)}_{\mathrm{fit}}$, variability in tissue composition ${\left(u_{B}\right)}_{\mathrm{comp}}$, and proton energy variation.

	${\left(u_{A}\right)}_{\mathrm{fit}}$ (%)		${\left(u_{B}\right)}_{\mathrm{comp}}$ (%)	
Energy (MeV)	Soft tissue	Bone tissue	Soft tissue	Bone tissue
70	2.49	0.94	4.57	1.66
100	2.51	0.94	4.60	1.73
150	2.53	0.96	4.63	1.80
230	2.54	0.99	4.65	1.87

### 3.4 Uncertainty in measured *SNR*

**FIGURE 7:**
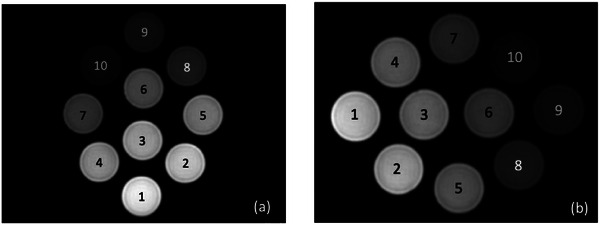
Fast spin‐echo (FSE)‐based proton density‐weighted MRI of the D_2_O‐H_2_O phantom acquired after (a) a 90° rotation and (b) repositioning at the couch edge.

Because the FSE‐based sequence provided a higher image quality, it was used to evaluate the positional uncertainty in *SNR*. Figure 7a,b show MR images of the D_2_O‐H_2_O phantom acquired after 90° rotation and repositioning at the edge of the couch, respectively. The relative differences (%) between $SNR_{\mathrm{arb}}$ and $SNR_{\mathrm{pri}}$ are summarized in Table 5. The uncertainty ${\left(u_{A}\right)}_{\mathrm{pos}}$ was 0.70% for 90° rotation and 1.24% for the edge of the couch position.

**TABLE 5:** Positional uncertainty ${\left(u_{A}\right)}_{\mathrm{pos}}$ in the signal‐noise ratio (SNR) measured by fast spin‐echo (FSE)‐based proton density‐weighted MRI.

#	Relative difference (%)	
	90° rotation	Couch edge position
1	−0.12	1.18
2	0.35	1.33
3	−1.00	1.13
4	0.64	−1.20
5	−0.13	1.25
6	−0.95	1.15
7	0.32	−1.27
8	0.47	−1.24
9	0.97	1.09
10	−1.10	1.50
${\left(u_{A}\right)}_{\mathrm{pos}}$	0.70	1.24

## 4 DISCUSSION

**FIGURE 8:**
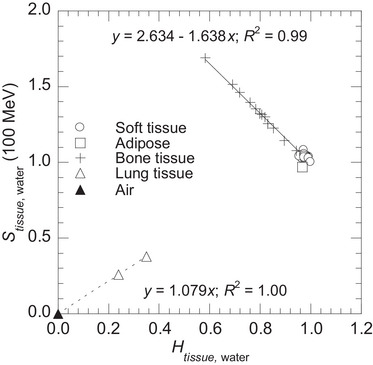
Relationship between tissue‐water hydrogen concentration ratio $H_{tissue,\;\mathrm{water}}$ and linear stopping power ratio $S_{tissue,\;\mathrm{water}}$, shown for air, inflated and compressed lungs (dotted line), and for soft and bone tissues (solid line).

As shown in Figure 3, the inflated lung was allocated to an area different from the soft and bone tissues. Although the inflated lung does not align with the linear regression line, its distinct $H_{tissue,\;\mathrm{water}}$ allows for the estimation of the reference $S_{tissue,\;\mathrm{water}}$. However, $S_{tissue,\;\mathrm{water}}$ may vary substantially in lung tissues due to the spatial distribution of air volume within the lung at a given respiratory state. Therefore, it is necessary to model the relationship between $H_{tissue,\;\mathrm{water}}$ and $S_{tissue,\;\mathrm{water}}$ using additional compositions that account for this variation. Kato et al.^31^ reported that lung CT numbers in healthy individuals generally range from −950 to −650 HU, corresponding to a mass density of approximately 0.05–0.35 g cm^−3^. ICRU 46^17^ provides composition of the inflated lung with a mass density of 0.26 g cm^−3^, while ICRP 110^21^ provides compositions of compressed lung and air, with mass densities of 0.38 g cm^−3^ and 0.001 g cm^−3^, respectively. Figure 8, a revised version of Figure 3, shows the dotted linear regression line representing the correlation (*R* ^2^ = 1.00) between $H_{tissue,\;\mathrm{water}}$ and $S_{tissue,\;\mathrm{water}}$ for air, inflated and compressed lungs. To evaluate the regression fit, variations of ± 2%, ± 10%, and ± 20% were applied to the mass density of the inflated lung with constant elemental composition. The elemental composition was assumed to be constant because changes in air volume during inhalation or exhalation affect mass density, while the elemental composition remains largely unchanged. This assumption is consistent with the reported composition of inflated, compressed and deflated lung tissues.^17^, ^21^, ^32^ Uncertainty from the regression fit ${\left(u_{A}\right)}_{\mathrm{fit}}$ was 0.05% (Equation 7). To evaluate variability in tissue composition, two representative tissue samples were modeled based on the component weight ratio (e.g., water, lipid) and mass density ranges reported in ICRP 23.^33^ Uncertainty due to variability in tissue composition ${\left(u_{B}\right)}_{\mathrm{comp}}$ was 0.66% (Equation 8). The compositions of air, inflated and compressed lung tissues, as well as the modeled tissue samples, are presented in Supplementary Material 1. Due to the limited number of lung tissue samples, we report degrees of freedom *v* to quantify reliability of the estimated uncertainties^34^. ${\left(u_{A}\right)}_{\mathrm{fit}}$ was derived from six samples with mass density variations up to ± 20%, corresponding to *v* = 5. For ${\left(u_{B}\right)}_{\mathrm{comp}}$, we conservatively assign *v* = 10, despite using only two samples, as they span the range of inflated lung tissue compositions reported in ICRP 23.^33^

**FIGURE 9:**
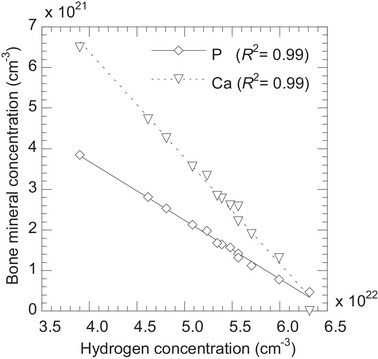
Relationship between the calcium and phosphorus concentrations, representing bone minerals, and hydrogen concentrations (mainly from water or lipid within bone tissues).

For bone tissues, $S_{tissue,\;\mathrm{water}}$ were closely aligned with the linear regression line (Figure 3), with an uncertainty ${\left(u_{A}\right)}_{\mathrm{fit}}$ of 0.94% at a proton energy of 100 MeV (Table 4). This phenomenon can be explained as follows. Bone tissues contain the mineral [hydroxyapatite (Ca_5_(PO_4_)_3_OH)], which mainly contains calcium and phosphorus.^17^, ^18^ Figure 9 shows the relationship between bone mineral and hydrogen concentrations (mainly from water or lipid). Linear correlations indicated that bone mineral concentration was inversely proportional to hydrogen concentration. Bone mineral has a high mass density of 3.225 g cm^−3^, which significantly influences $S_{tissue,\;\mathrm{water}}$. Since mineral concentration strongly influence $S_{tissue,\;\mathrm{water}}$ and is inversely proportional to hydrogen concentration, $S_{tissue,\;\mathrm{water}}$ could be accurately estimated using $H_{tissue,\;\mathrm{water}}$. In previous studies,^10^, ^13^, ^14^, ^16^ bone tissue was classified as either average spongiosa or cortical tissue. Our findings demonstrate that​ $S_{tissue,\;\mathrm{water}}$ can be estimated for all types of bone tissues reported in the ICRU 46^17^ using $H_{tissue,\;\mathrm{water}}$.

In contrast, most soft tissues contain water and lipid, which account for more than 70% of their total weight, and the remaining components are mainly proteins.^18^ The $H_{tissue,\;\mathrm{water}}$ for water and lipid are 1.00 and 0.97, respectively.^17^ Although the weight ratios of water and lipid varied among soft tissues, they did not significantly change $H_{tissue,\;\mathrm{water}}$. Therefore, the accurate determination of $S_{tissue,\;\mathrm{water}}$ using $H_{tissue,\;\mathrm{water}}$ is challenging, resulting in an uncertainty ${\left(u_{A}\right)}_{\mathrm{fit}}$ of 2.51% at a proton energy of 100 MeV (Table 4). However, in Figure 6a, the relative differences showed a different trend with $H_{tissue,\;\mathrm{water}}$, indicating that conducting a separate regression analysis for soft tissues could reduce uncertainties.

**FIGURE 10:**
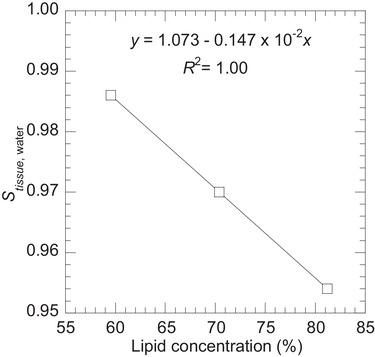
Relationship between lipid concentration and tissue‐water linear stopping power ratio $S_{tissue,\;\mathrm{water}}$ for adipose tissues.

As shown in Figures 3 and 6a, adipose tissue, which has a high lipid concentration, showed a significantly larger difference in $S_{tissue,\;\mathrm{water}}$ from the linear regression line. We analyzed whether any correlation existed between lipid concentration and $S_{tissue,\;\mathrm{water}}$ in adipose tissues^18^, as shown in Figure 10. An increase in lipid concentration reduces $S_{tissue,\;\mathrm{water}}$ owing to a corresponding decrease in mass density. The linear regression line represents the correlation between lipid concentration (%) and $S_{tissue,\;\mathrm{water}}$, with *R* ^2^ = 1.00. Accurate modeling of adipose tissue is important because it accounts for up to 19% of the total body weight in males and 26% in females.^33^ Therefore, conducting a separate measurement of lipid concentration is favorable. Excluding the relative difference in adipose tissue, uncertainty ${\left(u_{A}\right)}_{\mathrm{fit}}$ for the remaining soft tissues decreased to 1.70%. To estimate lipid concentration, chemical shift‐encoded MRI, which accurately estimates the proton density fat fraction regardless of the magnetic field strength, can be utilized.^35^ As both proton density‐weighted and chemical shift‐encoded MRI can be performed sequentially within the same imaging session and patient setup, no additional image registration is required. In voxels with a lipid concentration exceeding 50%, as the reported lipid concentration in adipose tissue ranges from 59.56%‒81.19%, ^18^ the correlation shown in Figure 10 can be applied to estimate $S_{tissue,\;\mathrm{water}}$. For other voxels, the relationship between $H_{tissue,\;\mathrm{water}}$ and $S_{tissue,\;\mathrm{water}}$ can be used. However, as this approach is beyond the scope of the present study, further studies are required to develop a precise workflow, potentially incorporating computational or machine learning techniques.

**FIGURE 11:**
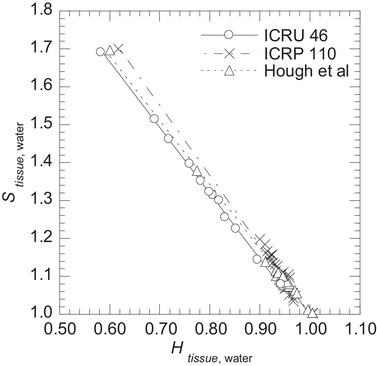
Relationship between tissue‐water hydrogen concentration ratio $H_{tissue,\;\mathrm{water}}$ and linear stopping power ratio $S_{tissue,\;\mathrm{water}}$ for bone tissue samples from ICRU 46^17^, ICRP 110^21^ and Hough et al.^29^ ^.^

For tissue samples with variable composition obtained from other references,^18^, ^21^, ^29^ the uncertainties ${\left(u_{B}\right)}_{\mathrm{comp}}$ were 4.60% for soft tissues and 1.73% for bone tissues (Table 4). The uncertainty in soft tissues increased due to variations in tissue composition. The limitations of the linear regression model and potential improvements for soft tissues have been discussed above. The increased uncertainty in bone tissues was mainly due to differences in the elemental composition of the cortical tissue [Supplementary Material 1]. Although the mass density remained consistent, $H_{tissue,\;\mathrm{water}}$ varied: 0.583 in ICRU 46,^17^ 0.617 in ICRP 110^21^ and 0.600 in Hough et al.^29^ Because bone tissues were modeled as mixtures of cortical tissue and bone marrow, such differences in cortical tissue affected the overall $H_{tissue,\;\mathrm{water}}$ of bone tissues. In our linear regression model, $H_{tissue,\;\mathrm{water}}$ is inversely proportional to $S_{tissue,\;\mathrm{water}}$, which is sensitive to variations in $H_{tissue,\;\mathrm{water}}$. As a result, bone tissue samples from other references 18, 21, 29 resulted in increased uncertainty compared to those from ICRU 46^17^. Figure 11 shows the relationship between $H_{tissue,\;\mathrm{water}}$ and $S_{tissue,\;\mathrm{water}}$ for bone tissue samples from ICRU 46,^17^ ICRP 110^21^ and Hough et al.^29^

The variation in proton energy from 70–230 MeV did not significantly affect the uncertainty. For soft tissues, the maximum uncertainty variation was 0.05% and for bone tissues, it was 0.14% at 230 MeV. No variation was observed in the uncertainty of the inflated lung.

The objective of our in‐house developed D_2_O‐H_2_O phantom was to accurately investigate the relationship between $H_{tissue,\;\mathrm{water}}$ and *SNR* measured by proton density‐weighted MRI, and to provide a simple and reproducible calibration tool. The phantom was successfully designed with $H_{solution,\;\mathrm{water}}$ ranging from 0.30 to 1.00, which closely approximates $H_{tissue,\;\mathrm{water}}$ (Table 1). In comparison between FSE and GRE‐based proton density‐weighted MRI, FSE‐based sequence provided a higher image quality and demonstrated a strong linear correlation between *SNR* and $H_{solution,\;\mathrm{water}}$ (Figure 4c), ensuring accurate estimation of hydrogen concentration. The GRE‐based sequence showed distortion artifacts due to its sensitivity to magnetic susceptibility variations in heterogeneous materials. In our phantom, the solutions were enclosed within PP containers, with air outside. Replacing the external air with water could improve the image quality. However, region such as lung or nasal cavity exhibits significant tissue heterogeneity, and the phantom design reflects similar anatomical conditions.

Due to positional uncertainty ${\left(u_{A}\right)}_{\mathrm{pos}}$ in the FSE‐based sequence, Type A uncertainty in estimated $S_{tissue,\;\mathrm{water}}$ for tissue samples from ICRU 46^17^ was 2.94% for soft, 1.39% for bone, and 1.00% for inflated lung tissues.

While ROIs were set to measure the mean voxel values of each D_2_O‐H_2_O solution to improve statistical accuracy, a voxel‐based approach is preferable for patient datasets as it preserves spatial heterogeneity and eliminates the need for ROI delineation. Therefore, it is also necessary to consider voxel‐level uncertainty in *SNR*. This uncertainty was categorized as Type A ${\left(u_{A}\right)}_{\mathrm{voxel}}$ and was estimated using the relative standard deviation $\sigma_{l,\mathrm{rel}}$, calculated as follows:

(9) $$\sigma_{l,\mathrm{rel}}=\frac{\sigma_{l}}{{\bar{I}}_{\mathrm{solution},\;l}}\;\times 100$$

where, $\sigma_{l}$ is the standard deviation of the mean voxel value ${\bar{I}}_{\mathrm{solution},\;l}$ in the $l^{\mathrm{th}}$ slice of the $k^{\mathrm{th}}$ D_2_O‐H_2_O solution. The RMS of $\sigma_{l,\mathrm{rel}}$ was calculated as described in Equation (7), with *N* = 150 (five slices per solution acquired at the primary, 90° rotated, and couch edge positions). The uncertainty ${\left(u_{A}\right)}_{\mathrm{voxel}}$ was 1.47%. Due to this voxel‐level uncertainty in *SNR*, the uncertainties in estimated $S_{tissue,\;\mathrm{water}}$ for tissue samples from ICRU 46^17^ was 3.38% for soft, 1.77% for bone, and 1.50% for inflated lung tissues.

Figure 5 shows the partial molar volume effect in D_2_O‐H_2_O solutions, which has not been previously reported. This finding demonstrates the importance of directly measuring the mass density, rather than calculating it, to avoid uncertainties associated with the partial molar volume when preparing similar phantoms.

To estimate lipid concentration using chemical shift‐encoded MRI, additional lipid concentrations representative of adipose tissue is required in the D_2_O‐H_2_O phantom. A simple way to do this is by preparing adipose tissue substitutes through mixing water, oil (lipid) and gelatin (protein) in different weight ratios.^36^

Table 6 presents the composite uncertainty $u_{C}$, calculated as the root sum square of all uncertainties analyzed in this study. Uncertainty sources such as intra or inter‐scanner *SNR* variability were not included, as the D_2_O‐H_2_O phantom was designed for accurate *SNR* calibration. $u_{C}$ were 6.89% for soft tissues, 3.00% for bone tissues, and 1.92% for the inflated lung. To interpret these results, a comparison was made with uncertainties reported for the CT‐based method: 1.6% for soft tissues, 2.4% for bone tissues, and 5.0% for lung tissues.^15^ For soft tissues, $u_{C}$ was larger with our method. For bone tissues, $u_{C}$ estimated using our method and CT‐based method was relatively similar. For lung tissues, the CT‐based method showed larger uncertainty, mainly due to the stoichiometric calibration, which estimates theoretical CT numbers of human tissues using tissue substitutes. Despite the limited samples for certain uncertainty criteria in our method, the effective degree of freedom for the composite lung uncertainty was calculated as $v_{\mathrm{eff}}=$128.71.^34^ This accounts for the degrees of freedom from regression fit (*v* = 5), variability in tissue composition (*v* = 10), positional uncertainty in FSE (*v* = 19), and voxel‐level uncertainty in *SNR* (*v* = 149), resulting in a statistically robust overall uncertainty estimate.

**TABLE 6:** Composite uncertainty $u_{C}$ in estimated tissue‐water linear stopping power ratio $S_{tissue,\;\mathrm{water}}$ using fast spin‐echo (FSE)‐based proton density‐weighted MRI.

	Uncertainty in estimated $S_{tissue,\;\mathrm{water}}$ (%)		
Uncertainty criteria	Soft tissue	Bone tissue	Inflated Lung
Linear regression fit (Type A)	2.51	0.94	0.05
Variability in tissue composition (Type B)	4.60	1.73	0.66
Sensitivity to proton energy variation	0.05	0.14	0.00
Positional uncertainty in FSE (Type A)	2.94	1.39	1.00
Voxel‐level uncertainty in *SNR* (Type A)	3.38	1.77	1.50
**Composite uncertainty** $u_{C}$	**6.89**	**3.00**	**1.92**

In addition to the uncertainties discussed above, we briefly address another potential source of uncertainty of mass density. Although this aspect is not clearly documented in the literature and was therefore excluded from our uncertainty analysis, noting it may help readers better understand the limitation of our method. In this study, the calculation of $H_{tissue,\;\mathrm{water}}$ and $S_{tissue,\;\mathrm{water}}$ relied on mass densities from ICRU 46.^17^ Woodard and White^18^ reported that these calculated mass densities were typically within ± 2% of the mean mass densities derived from published measured values. However, it is unclear whether the elemental composition of the compared tissue samples was identical. Since the linear relationship between $H_{tissue,\;\mathrm{water}}$ and $S_{tissue,\;\mathrm{water}}$ is sensitive to variations in mass density alone, deviations in actual mass density without changes in elemental composition would introduce an additional uncertainty. For instance, a ± 2% variation in mass density would correspond to an additional Type B uncertainty of 4.83% for soft, 3.27% for bone, and 0.05% for lung tissues, leading to increases in $u_{C}$ to 8.42%, 4.44%, and 1.92%, respectively. Nevertheless, as mentioned, no systematic estimate of such uncertainty in the reported mass density values exists in the literature. Examining this uncertainty would require experimental validation and is beyond the scope of the present study.

## 5 CONCLUSION

We evaluated the potential of proton density‐weighted MRI for estimating $S_{tissue,\;\mathrm{water}}$. Our results demonstrated that the relationship between $H_{tissue,\;\mathrm{water}}$ and $S_{tissue,\;\mathrm{water}}$ remains consistent despite variability in tissue composition and treatment energies. Our in‐house developed D_2_O‐H_2_O phantom demonstrated the feasibility of accurately calibrating proton density‐weighted MRI against $H_{tissue,\;\mathrm{water}}$ using an FSE‐based sequence. Based on these findings, proton density‐weighted MRI shows strong potential for estimating $S_{tissue,\;\mathrm{water}}$ and thereby facilitating MRI‐integrated proton therapy treatment planning. Additionally, because $H_{tissue,\;\mathrm{water}}$ is independent of the magnetic field strength, relationship between $H_{tissue,\;\mathrm{water}}$ and $S_{tissue,\;\mathrm{water}}$ is valid across different MRI systems. Future work will focus on integrating the $S_{tissue,\;\mathrm{water}}$ estimation for adipose tissues through lipid concentration measurement and establishing a clinically applicable workflow.

## CONFLICT OF INTEREST STATEMENT

The authors declare no conflicts of interest.

## Supporting information



Supporting Information

## Data Availability

The tissue compositions used in this study are presented in Supplementary Material 1.
