# Supplementary material for: Estimation of tissue‐water linear stopping power ratio of the proton beam from proton density‐weighted MRI
Source: Med Phys. 2025 Dec 27;53(1):e70204. doi: 10.1002/mp.70204 (PMC12743598; doi:10.1002/mp.70204)
Supplement: Supplementary file 1 — Supporting Information [file MP-53-0-s001.pdf]

**Supporting information**

# Estimation of tissue-water linear stopping power ratio of the proton beam from proton density-weighted MRI

**Puspen Chakraborty[1], Hidetoshi Saitoh[1], Junichi Hata[1], Weishan Chang[1]**

[1]*Graduate School of Human Health Sciences, Tokyo Metropolitan University, 7-2-10 Higashi-ogu, Arakawa-ku, Tokyo, Japan 116-8551*

## 1. Tissue composition for soft tissues

| # | Tissue samples (Adult) | Weight ratio | | | | | | | | | | | | | Mass density $\rho$ (g cm$^{-3}$) |
|---|---|---|---|---|---|---|---|---|---|---|---|---|---|---|---|
| | | H | C | N | O | Na | Mg | P | S | Cl | K | Ca | Fe | I | |
| | | | | | | | **ICRU report 46** [1] | | | | | | | | |
| 1 | Adipose | 0.114 | 0.598 | 0.007 | 0.278 | 0.001 | 0.000 | 0.000 | 0.001 | 0.001 | 0.000 | 0.000 | 0.000 | 0.000 | 0.950 |
| 2 | Mammary gland | 0.106 | 0.332 | 0.030 | 0.527 | 0.001 | 0.000 | 0.001 | 0.002 | 0.001 | 0.000 | 0.000 | 0.000 | 0.000 | 1.020 |
| 3 | Blood (whole) | 0.102 | 0.110 | 0.033 | 0.745 | 0.001 | 0.000 | 0.001 | 0.002 | 0.003 | 0.002 | 0.000 | 0.001 | 0.000 | 1.060 |
| 4 | Brain (whole) | 0.104 | 0.103 | 0.027 | 0.756 | 0.001 | 0.000 | 0.002 | 0.002 | 0.002 | 0.003 | 0.000 | 0.000 | 0.000 | 1.040 |
| 5 | GI tract | 0.106 | 0.115 | 0.022 | 0.751 | 0.001 | 0.000 | 0.001 | 0.001 | 0.002 | 0.001 | 0.000 | 0.000 | 0.000 | 1.030 |
| 6 | Heart (empty) | 0.104 | 0.139 | 0.029 | 0.718 | 0.001 | 0.000 | 0.002 | 0.002 | 0.002 | 0.003 | 0.000 | 0.000 | 0.000 | 1.050 |
| 7 | Heart (blood filled) | 0.103 | 0.121 | 0.032 | 0.734 | 0.001 | 0.000 | 0.001 | 0.002 | 0.003 | 0.002 | 0.000 | 0.001 | 0.000 | 1.060 |
| 8 | Kidney | 0.103 | 0.132 | 0.030 | 0.724 | 0.002 | 0.000 | 0.002 | 0.002 | 0.002 | 0.002 | 0.001 | 0.000 | 0.000 | 1.050 |
| 9 | Liver | 0.102 | 0.139 | 0.030 | 0.716 | 0.002 | 0.000 | 0.003 | 0.003 | 0.002 | 0.003 | 0.000 | 0.000 | 0.000 | 1.060 |
| 10 | Lung (congested) | 0.105 | 0.083 | 0.023 | 0.779 | 0.002 | 0.000 | 0.001 | 0.002 | 0.003 | 0.002 | 0.000 | 0.000 | 0.000 | 1.040 |
| 11 | Muscle | 0.102 | 0.143 | 0.034 | 0.710 | 0.001 | 0.000 | 0.002 | 0.003 | 0.001 | 0.004 | 0.000 | 0.000 | 0.000 | 1.050 |
| 12 | Ovary | 0.105 | 0.093 | 0.024 | 0.768 | 0.002 | 0.000 | 0.002 | 0.002 | 0.002 | 0.002 | 0.000 | 0.000 | 0.000 | 1.050 |
| 13 | Pancreas | 0.106 | 0.169 | 0.022 | 0.694 | 0.002 | 0.000 | 0.002 | 0.001 | 0.002 | 0.002 | 0.000 | 0.000 | 0.000 | 1.040 |
| 14 | Skin | 0.100 | 0.204 | 0.042 | 0.645 | 0.002 | 0.000 | 0.001 | 0.002 | 0.003 | 0.001 | 0.000 | 0.000 | 0.000 | 1.090 |
| 15 | Spleen | 0.103 | 0.113 | 0.032 | 0.741 | 0.001 | 0.000 | 0.003 | 0.002 | 0.002 | 0.003 | 0.000 | 0.000 | 0.000 | 1.060 |

| | | | | | | | | | | | | | | | |
|---|---|---|---|---|---|---|---|---|---|---|---|---|---|---|---|
| 16 | Testis | 0.106 | 0.099 | 0.020 | 0.766 | 0.002 | 0.000 | 0.001 | 0.002 | 0.002 | 0.002 | 0.000 | 0.000 | 0.000 | 1.040 |
| 17 | Thyroid | 0.104 | 0.119 | 0.024 | 0.745 | 0.002 | 0.000 | 0.001 | 0.001 | 0.002 | 0.001 | 0.000 | 0.000 | 0.001 | 1.050 |
| 18 | Bladder (empty) | 0.105 | 0.096 | 0.026 | 0.761 | 0.002 | 0.000 | 0.002 | 0.002 | 0.003 | 0.003 | 0.000 | 0.000 | 0.000 | 1.040 |
| 19 | Bladder (filled) | 0.108 | 0.035 | 0.015 | 0.830 | 0.003 | 0.000 | 0.001 | 0.001 | 0.005 | 0.002 | 0.000 | 0.000 | 0.000 | 1.030 |
| | **Woodard et al.** [2] | | | | | | | | | | | | | | |
| 20 | Adipose 1 | 0.112 | 0.517 | 0.013 | 0.355 | 0.001 | 0.000 | 0.000 | 0.001 | 0.001 | 0.000 | 0.000 | 0.000 | 0.000 | 0.970 |
| 21 | Adipose 2 | 0.116 | 0.681 | 0.002 | 0.198 | 0.001 | 0.000 | 0.000 | 0.001 | 0.001 | 0.000 | 0.000 | 0.000 | 0.000 | 0.930 |
| 22 | Mammary gland 1 | 0.109 | 0.506 | 0.023 | 0.358 | 0.001 | 0.000 | 0.001 | 0.001 | 0.001 | 0.000 | 0.000 | 0.000 | 0.000 | 0.990 |
| 23 | Mammary gland 2 | 0.102 | 0.158 | 0.037 | 0.698 | 0.001 | 0.000 | 0.001 | 0.002 | 0.001 | 0.000 | 0.000 | 0.000 | 0.000 | 1.060 |
| 24 | Heart (empty) 1 | 0.103 | 0.175 | 0.031 | 0.681 | 0.001 | 0.000 | 0.002 | 0.002 | 0.002 | 0.003 | 0.000 | 0.000 | 0.000 | 1.050 |
| 25 | Heart (empty) 2 | 0.104 | 0.103 | 0.027 | 0.756 | 0.001 | 0.000 | 0.002 | 0.002 | 0.002 | 0.003 | 0.000 | 0.000 | 0.000 | 1.050 |
| 26 | Kidney 1 | 0.102 | 0.160 | 0.034 | 0.693 | 0.002 | 0.000 | 0.002 | 0.002 | 0.002 | 0.002 | 0.001 | 0.000 | 0.000 | 1.050 |
| 27 | Kidney 2 | 0.104 | 0.106 | 0.027 | 0.752 | 0.002 | 0.000 | 0.002 | 0.002 | 0.002 | 0.002 | 0.001 | 0.000 | 0.000 | 1.050 |
| 28 | Liver 1 | 0.103 | 0.156 | 0.027 | 0.701 | 0.002 | 0.000 | 0.003 | 0.003 | 0.002 | 0.003 | 0.000 | 0.000 | 0.000 | 1.050 |
| 29 | Liver 2 | 0.101 | 0.126 | 0.033 | 0.727 | 0.002 | 0.000 | 0.003 | 0.003 | 0.002 | 0.003 | 0.000 | 0.000 | 0.000 | 1.070 |
| 30 | Muscle 1 | 0.101 | 0.171 | 0.036 | 0.681 | 0.001 | 0.000 | 0.002 | 0.003 | 0.001 | 0.004 | 0.000 | 0.000 | 0.000 | 1.050 |
| 31 | Muscle 2 | 0.102 | 0.112 | 0.030 | 0.745 | 0.001 | 0.000 | 0.002 | 0.003 | 0.001 | 0.004 | 0.000 | 0.000 | 0.000 | 1.050 |
| 32 | Skin 1 | 0.100 | 0.250 | 0.046 | 0.594 | 0.002 | 0.000 | 0.001 | 0.003 | 0.003 | 0.001 | 0.000 | 0.000 | 0.000 | 1.090 |
| 33 | Skin 2 | 0.101 | 0.158 | 0.037 | 0.695 | 0.002 | 0.000 | 0.001 | 0.002 | 0.003 | 0.001 | 0.000 | 0.000 | 0.000 | 1.090 |
| | **ICRP 110** [3] | | | | | | | | | | | | | | |
| 34 | Skin | 0.100 | 0.199 | 0.042 | 0.650 | 0.002 | 0.000 | 0.001 | 0.002 | 0.003 | 0.001 | 0.000 | 0.000 | 0.000 | 1.090 |
| 35 | Liver | 0.102 | 0.130 | 0.031 | 0.725 | 0.002 | 0.000 | 0.002 | 0.003 | 0.002 | 0.003 | 0.000 | 0.000 | 0.000 | 1.050 |
| 36 | Pancreas (Male) | 0.105 | 0.155 | 0.025 | 0.706 | 0.002 | 0.000 | 0.002 | 0.001 | 0.002 | 0.002 | 0.000 | 0.000 | 0.000 | 1.050 |
| 37 | Pancreas (Female) | 0.105 | 0.157 | 0.024 | 0.705 | 0.002 | 0.000 | 0.002 | 0.001 | 0.002 | 0.002 | 0.000 | 0.000 | 0.000 | 1.050 |
| 38 | Brain (Male) | 0.107 | 0.095 | 0.018 | 0.767 | 0.002 | 0.000 | 0.003 | 0.002 | 0.003 | 0.003 | 0.000 | 0.000 | 0.000 | 1.040 |
| 39 | Brain (Female) | 0.107 | 0.144 | 0.022 | 0.713 | 0.002 | 0.000 | 0.004 | 0.002 | 0.003 | 0.003 | 0.000 | 0.000 | 0.000 | 1.040 |

| | | | | | | | | | | | | | | |
|---|---|---|---|---|---|---|---|---|---|---|---|---|---|---|
| 40 | Kidneys (Male) | 0.103 | 0.124 | 0.031 | 0.731 | 0.002 | 0.000 | 0.002 | 0.002 | 0.002 | 0.002 | 0.001 | 0.000 | 0.000 | 1.050 |
| 41 | Kidneys (Female) | 0.103 | 0.125 | 0.031 | 0.730 | 0.002 | 0.000 | 0.002 | 0.002 | 0.002 | 0.002 | 0.001 | 0.000 | 0.000 | 1.050 |
| 42 | Stomach | 0.105 | 0.114 | 0.025 | 0.750 | 0.001 | 0.000 | 0.001 | 0.001 | 0.002 | 0.001 | 0.000 | 0.000 | 0.000 | 1.040 |
| 43 | Intestine | 0.105 | 0.113 | 0.026 | 0.750 | 0.001 | 0.000 | 0.001 | 0.001 | 0.002 | 0.001 | 0.000 | 0.000 | 0.000 | 1.040 |
| 44 | Spleen (Male) | 0.102 | 0.111 | 0.033 | 0.743 | 0.001 | 0.000 | 0.002 | 0.002 | 0.003 | 0.002 | 0.000 | 0.001 | 0.000 | 1.040 |
| 45 | Spleen (Female) | 0.103 | 0.112 | 0.032 | 0.743 | 0.001 | 0.000 | 0.002 | 0.002 | 0.002 | 0.003 | 0.000 | 0.000 | 0.000 | 1.040 |
| 46 | Thyroid (Male) | 0.104 | 0.117 | 0.026 | 0.745 | 0.002 | 0.000 | 0.001 | 0.001 | 0.002 | 0.001 | 0.000 | 0.000 | 0.001 | 1.040 |
| 47 | Thyroid (Female) | 0.104 | 0.118 | 0.025 | 0.745 | 0.002 | 0.000 | 0.001 | 0.001 | 0.002 | 0.001 | 0.000 | 0.000 | 0.001 | 1.040 |
| 48 | Testis | 0.106 | 0.100 | 0.021 | 0.764 | 0.002 | 0.000 | 0.001 | 0.002 | 0.002 | 0.002 | 0.000 | 0.000 | 0.000 | 1.040 |
| 49 | Ovaries | 0.105 | 0.094 | 0.025 | 0.766 | 0.002 | 0.000 | 0.002 | 0.002 | 0.002 | 0.002 | 0.000 | 0.000 | 0.000 | 1.040 |
| 50 | Adrenal (Male) | 0.104 | 0.221 | 0.028 | 0.637 | 0.001 | 0.000 | 0.002 | 0.003 | 0.002 | 0.002 | 0.000 | 0.000 | 0.000 | 1.030 |
| 51 | Adrenal (Female) | 0.104 | 0.228 | 0.028 | 0.630 | 0.001 | 0.000 | 0.002 | 0.003 | 0.002 | 0.002 | 0.000 | 0.000 | 0.000 | 1.030 |
| 52 | Esophagus (Male) | 0.104 | 0.213 | 0.029 | 0.644 | 0.001 | 0.000 | 0.002 | 0.003 | 0.002 | 0.002 | 0.000 | 0.000 | 0.000 | 1.030 |
| 53 | Esophagus (Female) | 0.104 | 0.222 | 0.028 | 0.636 | 0.001 | 0.000 | 0.002 | 0.003 | 0.002 | 0.002 | 0.000 | 0.000 | 0.000 | 1.030 |
| 54 | Gallbladder [a] (Male) | 0.104 | 0.231 | 0.028 | 0.627 | 0.001 | 0.000 | 0.002 | 0.003 | 0.002 | 0.002 | 0.000 | 0.000 | 0.000 | 1.030 |
| 55 | Gallbladder [a] (Female) | 0.105 | 0.235 | 0.028 | 0.622 | 0.001 | 0.000 | 0.002 | 0.003 | 0.002 | 0.002 | 0.000 | 0.000 | 0.000 | 1.030 |
| 56 | Prostate | 0.104 | 0.231 | 0.028 | 0.627 | 0.001 | 0.000 | 0.002 | 0.003 | 0.002 | 0.002 | 0.000 | 0.000 | 0.000 | 1.030 |
| 57 | Uterus | 0.105 | 0.286 | 0.025 | 0.576 | 0.001 | 0.000 | 0.002 | 0.002 | 0.001 | 0.002 | 0.000 | 0.000 | 0.000 | 1.030 |
| 58 | Urine | 0.107 | 0.003 | 0.010 | 0.873 | 0.004 | 0.000 | 0.001 | 0.000 | 0.000 | 0.002 | 0.000 | 0.000 | 0.000 | 1.040 |

[a] In ICRP 110, the tissue compositions of the pituitary, trachea, thymus, tonsils, ureters are assumed to be the same as that of the gallbladder.

## 2. Tissue composition for bone tissues

| # | Tissue samples (Adult) | Weight ratio | | | | | | | | | | | | | Mass density |
|---|---|---|---|---|---|---|---|---|---|---|---|---|---|---|---|
| | | H | C | N | O | Na | Mg | P | S | Cl | K | Ca | Fe | I | $\rho$ (g $cm^{-3}$) |
| | **ICRU report 46** [1] | | | | | | | | | | | | | | |
| 1 | Cartilage | 0.096 | 0.099 | 0.022 | 0.744 | 0.005 | 0.000 | 0.022 | 0.009 | 0.003 | 0.000 | 0.000 | 0.000 | 0.000 | 1.100 |
| 2 | Cortical bone | 0.034 | 0.155 | 0.042 | 0.435 | 0.001 | 0.002 | 0.103 | 0.003 | 0.000 | 0.000 | 0.225 | 0.000 | 0.000 | 1.920 |
| 3 | Cranium (whole) | 0.050 | 0.212 | 0.040 | 0.435 | 0.001 | 0.002 | 0.081 | 0.003 | 0.000 | 0.000 | 0.176 | 0.000 | 0.000 | 1.610 |
| 4 | Femur (whole)_30 years | 0.070 | 0.345 | 0.028 | 0.368 | 0.001 | 0.001 | 0.055 | 0.002 | 0.001 | 0.000 | 0.129 | 0.000 | 0.000 | 1.330 |
| 5 | Humerus (whole) | 0.060 | 0.314 | 0.031 | 0.369 | 0.001 | 0.001 | 0.070 | 0.002 | 0.000 | 0.000 | 0.152 | 0.000 | 0.000 | 1.460 |
| 6 | Mandible (whole) | 0.046 | 0.199 | 0.041 | 0.435 | 0.001 | 0.002 | 0.086 | 0.003 | 0.000 | 0.000 | 0.187 | 0.000 | 0.000 | 1.680 |
| 7 | Ribs (whole)_$2^{nd}$, $6^{th}$ | 0.064 | 0.263 | 0.039 | 0.436 | 0.001 | 0.001 | 0.060 | 0.003 | 0.001 | 0.001 | 0.131 | 0.000 | 0.000 | 1.410 |
| 8 | Ribs (whole)_$10^{th}$ | 0.056 | 0.235 | 0.040 | 0.434 | 0.001 | 0.001 | 0.072 | 0.003 | 0.001 | 0.001 | 0.156 | 0.000 | 0.000 | 1.520 |
| 9 | Sacrum (whole)_Male | 0.074 | 0.302 | 0.037 | 0.438 | 0.000 | 0.001 | 0.045 | 0.002 | 0.001 | 0.001 | 0.098 | 0.001 | 0.000 | 1.290 |
| 10 | Sacrum (whole)_Female | 0.066 | 0.271 | 0.038 | 0.435 | 0.001 | 0.001 | 0.058 | 0.003 | 0.001 | 0.001 | 0.125 | 0.000 | 0.000 | 1.390 |
| 11 | Spongiosa | 0.085 | 0.404 | 0.028 | 0.367 | 0.001 | 0.001 | 0.034 | 0.002 | 0.002 | 0.001 | 0.074 | 0.001 | 0.000 | 1.180 |
| 12 | Vertebra (whole)_C4 | 0.063 | 0.261 | 0.039 | 0.436 | 0.001 | 0.001 | 0.061 | 0.003 | 0.001 | 0.001 | 0.133 | 0.000 | 0.000 | 1.420 |
| 13 | Vertebra (whole)_D6, L3 | 0.070 | 0.287 | 0.038 | 0.437 | 0.000 | 0.001 | 0.051 | 0.002 | 0.001 | 0.001 | 0.111 | 0.001 | 0.000 | 1.330 |
| | **ICRP 110** [3] | | | | | | | | | | | | | | |
| 14 | Cortical bone | 0.036 | 0.159 | 0.042 | 0.448 | 0.003 | 0.002 | 0.094 | 0.003 | 0.000 | 0.000 | 0.213 | 0.000 | 0.000 | 1.920 |
| 15 | Humerus, Upper half (Male) | 0.085 | 0.288 | 0.026 | 0.498 | 0.002 | 0.001 | 0.033 | 0.004 | 0.002 | 0.000 | 0.061 | 0.000 | 0.000 | 1.205 |
| 16 | Humerus, Upper half (Female) | 0.087 | 0.366 | 0.025 | 0.422 | 0.002 | 0.001 | 0.030 | 0.003 | 0.001 | 0.001 | 0.062 | 0.000 | 0.000 | 1.185 |

| | | | | | | | | | | | | | | | |
|---|---|---|---|---|---|---|---|---|---|---|---|---|---|---|---|
| 17 | Humerus, Lower half (Male) | 0.097 | 0.439 | 0.017 | 0.381 | 0.002 | 0.000 | 0.021 | 0.003 | 0.001 | 0.000 | 0.039 | 0.000 | 0.000 | 1.108 |
| 18 | Humerus, Lower half (Female) | 0.096 | 0.473 | 0.017 | 0.341 | 0.002 | 0.000 | 0.022 | 0.002 | 0.001 | 0.000 | 0.046 | 0.000 | 0.000 | 1.117 |
| 19 | Clavicle (Male) | 0.091 | 0.348 | 0.024 | 0.457 | 0.002 | 0.000 | 0.026 | 0.003 | 0.001 | 0.000 | 0.048 | 0.000 | 0.000 | 1.151 |
| 20 | Clavicle (Female) | 0.087 | 0.361 | 0.025 | 0.424 | 0.002 | 0.001 | 0.031 | 0.003 | 0.001 | 0.001 | 0.064 | 0.000 | 0.000 | 1.191 |
| 21 | Cranium (Male) | 0.090 | 0.335 | 0.025 | 0.467 | 0.002 | 0.000 | 0.026 | 0.003 | 0.002 | 0.001 | 0.049 | 0.000 | 0.000 | 1.157 |
| 22 | Cranium (Female) | 0.081 | 0.317 | 0.028 | 0.451 | 0.002 | 0.001 | 0.037 | 0.003 | 0.001 | 0.001 | 0.078 | 0.000 | 0.000 | 1.245 |
| 23 | Femur, Upper half (Male) | 0.094 | 0.385 | 0.022 | 0.430 | 0.002 | 0.000 | 0.022 | 0.003 | 0.001 | 0.000 | 0.041 | 0.000 | 0.000 | 1.124 |
| 24 | Femur, Upper half (Female) | 0.104 | 0.496 | 0.018 | 0.349 | 0.001 | 0.000 | 0.009 | 0.002 | 0.001 | 0.001 | 0.019 | 0.000 | 0.000 | 1.046 |
| 25 | Mandible (Male) | 0.083 | 0.266 | 0.027 | 0.511 | 0.003 | 0.001 | 0.036 | 0.004 | 0.002 | 0.000 | 0.067 | 0.000 | 0.000 | 1.228 |
| 26 | Mandible (Female) | 0.087 | 0.357 | 0.026 | 0.429 | 0.002 | 0.001 | 0.030 | 0.003 | 0.001 | 0.001 | 0.063 | 0.000 | 0.000 | 1.189 |
| 27 | Pelvic bone (Male) | 0.094 | 0.360 | 0.025 | 0.454 | 0.002 | 0.000 | 0.021 | 0.003 | 0.002 | 0.001 | 0.038 | 0.000 | 0.000 | 1.123 |
| 28 | Pelvic bone (Female) | 0.096 | 0.406 | 0.025 | 0.412 | 0.001 | 0.000 | 0.018 | 0.002 | 0.001 | 0.001 | 0.038 | 0.000 | 0.000 | 1.109 |
| 29 | Ribs (Male) | 0.089 | 0.292 | 0.029 | 0.507 | 0.002 | 0.000 | 0.026 | 0.004 | 0.002 | 0.001 | 0.048 | 0.000 | 0.000 | 1.165 |
| 30 | Ribs (Female) | 0.097 | 0.381 | 0.028 | 0.445 | 0.001 | 0.000 | 0.014 | 0.002 | 0.002 | 0.001 | 0.028 | 0.001 | 0.000 | 1.092 |
| 31 | Scapula (Male) | 0.087 | 0.309 | 0.026 | 0.483 | 0.002 | 0.001 | 0.030 | 0.004 | 0.002 | 0.000 | 0.056 | 0.000 | 0.000 | 1.183 |
| 32 | Scapula (Female) | 0.094 | 0.406 | 0.024 | 0.404 | 0.001 | 0.000 | 0.022 | 0.002 | 0.001 | 0.001 | 0.045 | 0.000 | 0.000 | 1.128 |
| 33 | Cervical spine (Male) | 0.103 | 0.309 | 0.026 | 0.483 | 0.002 | 0.001 | 0.030 | 0.004 | 0.002 | 0.000 | 0.056 | 0.000 | 0.000 | 1.050 |
| 34 | Cervical spine (Female) | 0.092 | 0.351 | 0.029 | 0.458 | 0.001 | 0.000 | 0.021 | 0.002 | 0.002 | 0.001 | 0.043 | 0.000 | 0.000 | 1.135 |
| 35 | Thoracic spine (Male) | 0.099 | 0.376 | 0.027 | 0.459 | 0.001 | 0.000 | 0.012 | 0.002 | 0.002 | 0.001 | 0.020 | 0.001 | 0.000 | 1.074 |
| 36 | Thoracic spine (Female) | 0.098 | 0.386 | 0.028 | 0.442 | 0.001 | 0.000 | 0.013 | 0.002 | 0.002 | 0.001 | 0.026 | 0.001 | 0.000 | 1.084 |
| 37 | Lumbar spine (Male) | 0.095 | 0.340 | 0.028 | 0.480 | 0.001 | 0.000 | 0.018 | 0.003 | 0.002 | 0.001 | 0.032 | 0.000 | 0.000 | 1.112 |
| 38 | Lumbar spine (Female) | 0.088 | 0.329 | 0.030 | 0.466 | 0.001 | 0.001 | 0.026 | 0.003 | 0.001 | 0.001 | 0.054 | 0.000 | 0.000 | 1.171 |
| 39 | Sacrum (Male) | 0.105 | 0.419 | 0.027 | 0.432 | 0.001 | 0.000 | 0.004 | 0.002 | 0.002 | 0.001 | 0.006 | 0.001 | 0.000 | 1.031 |
| 40 | Sacrum (Female) | 0.102 | 0.410 | 0.027 | 0.433 | 0.001 | 0.000 | 0.007 | 0.002 | 0.002 | 0.001 | 0.014 | 0.001 | 0.000 | 1.052 |

| | | | | | | | | | | | | | | | |
|---|---|---|---|---|---|---|---|---|---|---|---|---|---|---|---|
| 41 | Sternum (Male) | 0.104 | 0.409 | 0.027 | 0.438 | 0.001 | 0.000 | 0.006 | 0.002 | 0.002 | 0.001 | 0.009 | 0.001 | 0.000 | 1.041 |
| 42 | Sternum (Female) | 0.099 | 0.392 | 0.028 | 0.439 | 0.001 | 0.000 | 0.012 | 0.002 | 0.002 | 0.001 | 0.023 | 0.001 | 0.000 | 1.076 |
| 43 | Medulary cavity [b] (Male) | 0.115 | 0.636 | 0.007 | 0.239 | 0.001 | 0.000 | 0.000 | 0.001 | 0.001 | 0.001 | 0.000 | 0.000 | 0.000 | 0.980 |
| 44 | Medulary cavity [b] (Female) | 0.115 | 0.637 | 0.007 | 0.238 | 0.001 | 0.000 | 0.000 | 0.001 | 0.001 | 0.000 | 0.000 | 0.000 | 0.000 | 0.980 |
| | **Hough et al.** [4] | | | | | | | | | | | | | | |
| 45 | Cortical bone | 0.035 | 0.160 | 0.042 | 0.445 | 0.003 | 0.002 | 0.095 | 0.003 | 0.000 | 0.000 | 0.215 | 0.000 | 0.000 | 1.920 |
| 46 | Craniofacial bone | 0.058 | 0.274 | 0.035 | 0.410 | 0.002 | 0.002 | 0.067 | 0.003 | 0.000 | 0.000 | 0.150 | 0.000 | 0.000 | 1.492 |
| 47 | Mandible | 0.099 | 0.486 | 0.022 | 0.341 | 0.001 | 0.001 | 0.016 | 0.002 | 0.000 | 0.000 | 0.033 | 0.000 | 0.000 | 1.077 |
| 48 | Scapula | 0.092 | 0.448 | 0.024 | 0.353 | 0.002 | 0.001 | 0.025 | 0.002 | 0.000 | 0.000 | 0.054 | 0.000 | 0.000 | 1.134 |
| 49 | Clavicle | 0.097 | 0.479 | 0.022 | 0.338 | 0.001 | 0.001 | 0.020 | 0.002 | 0.000 | 0.000 | 0.042 | 0.000 | 0.000 | 1.099 |
| 50 | Sternum | 0.098 | 0.430 | 0.028 | 0.394 | 0.001 | 0.002 | 0.015 | 0.002 | 0.000 | 0.000 | 0.030 | 0.001 | 0.000 | 1.086 |
| 51 | Ribs | 0.094 | 0.412 | 0.029 | 0.398 | 0.001 | 0.002 | 0.020 | 0.002 | 0.000 | 0.000 | 0.042 | 0.001 | 0.000 | 1.118 |
| 52 | Cervical vertebra | 0.087 | 0.385 | 0.031 | 0.403 | 0.002 | 0.002 | 0.028 | 0.002 | 0.000 | 0.000 | 0.061 | 0.001 | 0.000 | 1.169 |
| 53 | Thoracic vertebra | 0.096 | 0.424 | 0.029 | 0.395 | 0.001 | 0.002 | 0.017 | 0.002 | 0.000 | 0.000 | 0.034 | 0.001 | 0.000 | 1.097 |
| 54 | Lumbar vertebra | 0.095 | 0.419 | 0.029 | 0.396 | 0.001 | 0.002 | 0.018 | 0.002 | 0.000 | 0.000 | 0.037 | 0.001 | 0.000 | 1.105 |
| 55 | Sacrum | 0.094 | 0.412 | 0.029 | 0.398 | 0.001 | 0.002 | 0.020 | 0.002 | 0.000 | 0.000 | 0.042 | 0.001 | 0.000 | 1.117 |
| 56 | Pelvic bone | 0.097 | 0.461 | 0.024 | 0.360 | 0.001 | 0.001 | 0.017 | 0.002 | 0.000 | 0.000 | 0.036 | 0.000 | 0.000 | 1.091 |
| 57 | Humerus, proximal | 0.100 | 0.506 | 0.019 | 0.320 | 0.001 | 0.001 | 0.017 | 0.002 | 0.000 | 0.000 | 0.035 | 0.000 | 0.000 | 1.078 |
| 58 | Humerus, upper shaft | 0.112 | 0.575 | 0.014 | 0.294 | 0.001 | 0.001 | 0.001 | 0.001 | 0.000 | 0.000 | 0.000 | 0.000 | 0.000 | 0.994 |
| 59 | Humerus, lower shaft | 0.115 | 0.632 | 0.008 | 0.242 | 0.001 | 0.000 | 0.001 | 0.001 | 0.000 | 0.000 | 0.000 | 0.000 | 0.000 | 0.981 |
| 60 | Humerus, distal | 0.095 | 0.513 | 0.016 | 0.294 | 0.002 | 0.001 | 0.025 | 0.002 | 0.000 | 0.000 | 0.054 | 0.000 | 0.000 | 1.117 |
| 61 | Radius, proximal | 0.102 | 0.559 | 0.013 | 0.274 | 0.001 | 0.000 | 0.016 | 0.001 | 0.000 | 0.000 | 0.033 | 0.000 | 0.000 | 1.061 |
| 62 | Radius, shaft | 0.115 | 0.632 | 0.008 | 0.242 | 0.001 | 0.000 | 0.001 | 0.001 | 0.000 | 0.000 | 0.000 | 0.000 | 0.000 | 0.981 |
| 63 | Radius, distal | 0.099 | 0.538 | 0.014 | 0.283 | 0.001 | 0.000 | 0.020 | 0.001 | 0.000 | 0.000 | 0.042 | 0.000 | 0.000 | 1.085 |
| 64 | Ulna, proximal | 0.094 | 0.506 | 0.017 | 0.297 | 0.002 | 0.001 | 0.026 | 0.002 | 0.000 | 0.000 | 0.057 | 0.000 | 0.000 | 1.125 |
| 65 | Ulna, shaft | 0.115 | 0.632 | 0.008 | 0.242 | 0.001 | 0.000 | 0.001 | 0.001 | 0.000 | 0.000 | 0.000 | 0.000 | 0.000 | 0.981 |

| | | | | | | | | | | | | | | | |
|---|---|---|---|---|---|---|---|---|---|---|---|---|---|---|---|
| 66 | Ulna, distal | 0.096 | 0.521 | 0.016 | 0.291 | 0.002 | 0.001 | 0.023 | 0.002 | 0.000 | 0.000 | 0.050 | 0.000 | 0.000 | 1.106 |
| 67 | Wrist and hand bone | 0.095 | 0.513 | 0.016 | 0.294 | 0.002 | 0.001 | 0.025 | 0.002 | 0.000 | 0.000 | 0.054 | 0.000 | 0.000 | 1.117 |
| 68 | Femur, proximal | 0.093 | 0.472 | 0.021 | 0.333 | 0.002 | 0.001 | 0.024 | 0.002 | 0.000 | 0.000 | 0.053 | 0.000 | 0.000 | 1.126 |
| 69 | Femur, upper shaft | 0.112 | 0.575 | 0.014 | 0.294 | 0.001 | 0.001 | 0.001 | 0.001 | 0.000 | 0.000 | 0.000 | 0.000 | 0.000 | 0.994 |
| 70 | Femur, lower shaft | 0.115 | 0.632 | 0.008 | 0.242 | 0.001 | 0.000 | 0.001 | 0.001 | 0.000 | 0.000 | 0.000 | 0.000 | 0.000 | 0.981 |
| 71 | Femur, distal | 0.095 | 0.516 | 0.016 | 0.293 | 0.002 | 0.001 | 0.024 | 0.002 | 0.000 | 0.000 | 0.052 | 0.000 | 0.000 | 1.112 |
| 72 | Patella | 0.095 | 0.516 | 0.016 | 0.293 | 0.002 | 0.001 | 0.024 | 0.002 | 0.000 | 0.000 | 0.052 | 0.000 | 0.000 | 1.112 |
| 73 | Tibia, proximal | 0.099 | 0.538 | 0.014 | 0.283 | 0.001 | 0.000 | 0.020 | 0.001 | 0.000 | 0.000 | 0.042 | 0.000 | 0.000 | 1.085 |
| 74 | Tibia, shaft | 0.115 | 0.632 | 0.008 | 0.242 | 0.001 | 0.000 | 0.001 | 0.001 | 0.000 | 0.000 | 0.000 | 0.000 | 0.000 | 0.981 |
| 75 | Tibia, distal | 0.098 | 0.531 | 0.015 | 0.286 | 0.001 | 0.000 | 0.021 | 0.002 | 0.000 | 0.000 | 0.045 | 0.000 | 0.000 | 1.093 |
| 76 | Fibula, Proximal | 0.104 | 0.567 | 0.012 | 0.271 | 0.001 | 0.000 | 0.014 | 0.001 | 0.000 | 0.000 | 0.029 | 0.000 | 0.000 | 1.051 |
| 77 | Fibula, shaft | 0.115 | 0.632 | 0.008 | 0.242 | 0.001 | 0.000 | 0.001 | 0.001 | 0.000 | 0.000 | 0.000 | 0.000 | 0.000 | 0.981 |
| 78 | Fibula, distal | 0.095 | 0.516 | 0.016 | 0.293 | 0.002 | 0.001 | 0.024 | 0.002 | 0.000 | 0.000 | 0.052 | 0.000 | 0.000 | 1.113 |
| 79 | Ankle and foot bone | 0.095 | 0.516 | 0.016 | 0.293 | 0.002 | 0.001 | 0.024 | 0.002 | 0.000 | 0.000 | 0.052 | 0.000 | 0.000 | 1.112 |

[b] In ICRP 110, the tissue composition of the medullary cavity is assumed to be the same for the humerus, femur, lower arm and lower leg bones.

## 3. Tissue composition for inflated lung

| # | Tissue samples (Adult) | Weight ratio | | | | | | | | | | | | | Mass density |
|---|---|---|---|---|---|---|---|---|---|---|---|---|---|---|---|
| | | H | C | N | O | Na | Mg | P | S | Cl | K | Ca | Fe | I | $\rho$ (g $cm^{-3}$) |
| | | | | | | | **ICRU report 46** [1] | | | | | | | | |
| 1 | Inflated lung | 0.103 | 0.105 | 0.031 | 0.749 | 0.002 | 0.000 | 0.002 | 0.003 | 0.003 | 0.002 | 0.000 | 0.000 | 0.000 | 0.260 |
| | | | | | | | **ICRP 110** [3] | | | | | | | | |
| 2 | Compressed lung | 0.103 | 0.107 | 0.032 | 0.746 | 0.002 | 0.000 | 0.002 | 0.003 | 0.003 | 0.002 | 0.000 | 0.000 | 0.000 | 0.380 |
| 3 | Air | 0.000 | 0.000 | 0.800 | 0.200 | 0.000 | 0.000 | 0.000 | 0.000 | 0.000 | 0.000 | 0.000 | 0.000 | 0.000 | 0.001 |

| | | | | | | | | | | | | | | | |
|---|---|---|---|---|---|---|---|---|---|---|---|---|---|---|
| 4 | Inflated lung (Modeled 1) | 0.104 | 0.096 | 0.027 | 0.766 | 0.000 | 0.000 | 0.002 | 0.002 | 0.000 | 0.000 | 0.004 | 0.000 | 0.000 | 0.230 |
| 5 | Inflated lung (Modeled 2) | 0.101 | 0.118 | 0.035 | 0.736 | 0.000 | 0.000 | 0.003 | 0.002 | 0.000 | 0.000 | 0.006 | 0.000 | 0.000 | 0.290 |

## References


1. ICRU Report 46: Photon, Electron, Proton and Neutron Interaction Data for Body Tissues, International Commission on Radiation Units and Measurements, 1992.
2. Woodard HQ, White DR. The composition of body tissues. *Br J Radiol*. 1986;59(708):1209-1219. doi: 10.1259/0007-1285-59-708-1209.
3. Menzel HG, Clement C, DeLuca P. ICRP Publication 110, Realistic reference phantoms: A report of adult reference computational phantoms. *Ann ICRP*. 2009;39(2):1-164. doi: 10.1016/j.icrp.2009.09.001.
4. Hough M, Johnson P, Rajon D, Jokisch D, Lee C, Bolch W. An image-based skeletal dosimetry model for the ICRP reference adult male - Internal electron sources. *Phys Med Biol*. 2011;56(8):2309-2346. doi:10.1088/0031-9155/56/8/001.
